# Supplementary material for: Dual-specificity protein phosphatase 6 (DUSP6) overexpression reduces amyloid load and improves memory deficits in male 5xFAD mice
Source: Front Aging Neurosci. 2024 Jun 28;16:1400447. doi: 10.3389/fnagi.2024.1400447 (PMC11239576; doi:10.3389/fnagi.2024.1400447)
Supplement: Supplementary file 2 [file Data_Sheet_2.docx]

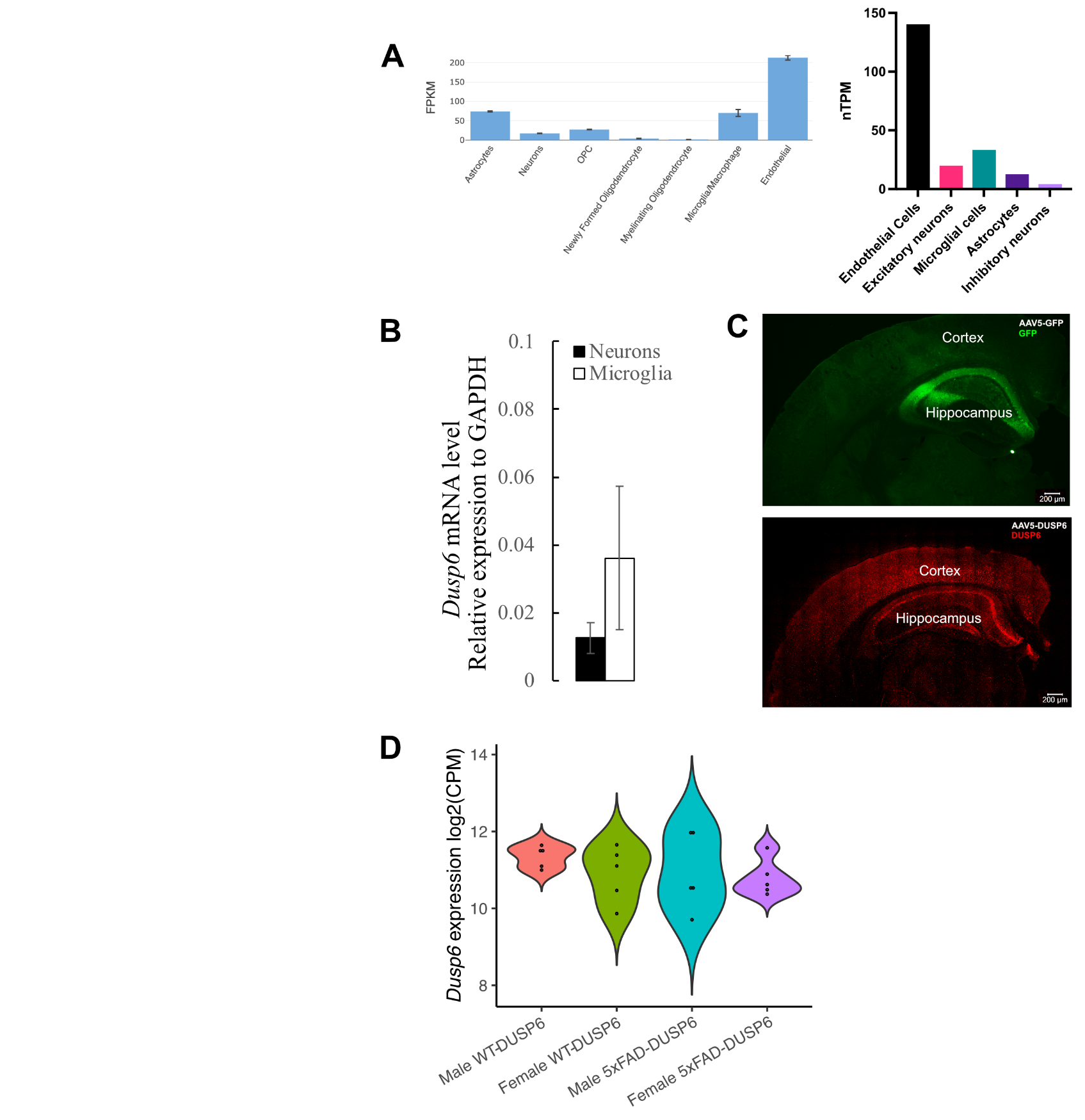


**Fig. S1.** *DUSP6* is expressed in both human iPSC-derived neurons and microglia. (A) Cell-type expression of *Dusp6* mRNA [source [www.brainrnaseq.org](http://www.brainrnaseq.org) (left plot)], and single cell RNAseq data of human *DUSP6* expression [source [www.proteinatlas.org](http://www.proteinatlas.org) (right plot)]. (B) qPCR expression analysis of *DUSP6* in hiPSC-derived cells, cortical neurons and microglial cells. (C) Representative images of coronal sections of hippocampus injected with AAV5-GFP (top) and AAV5-DUSP6 (bottom). (D) Violin plots showing the overexpression of *Dusp6* gene in each group.


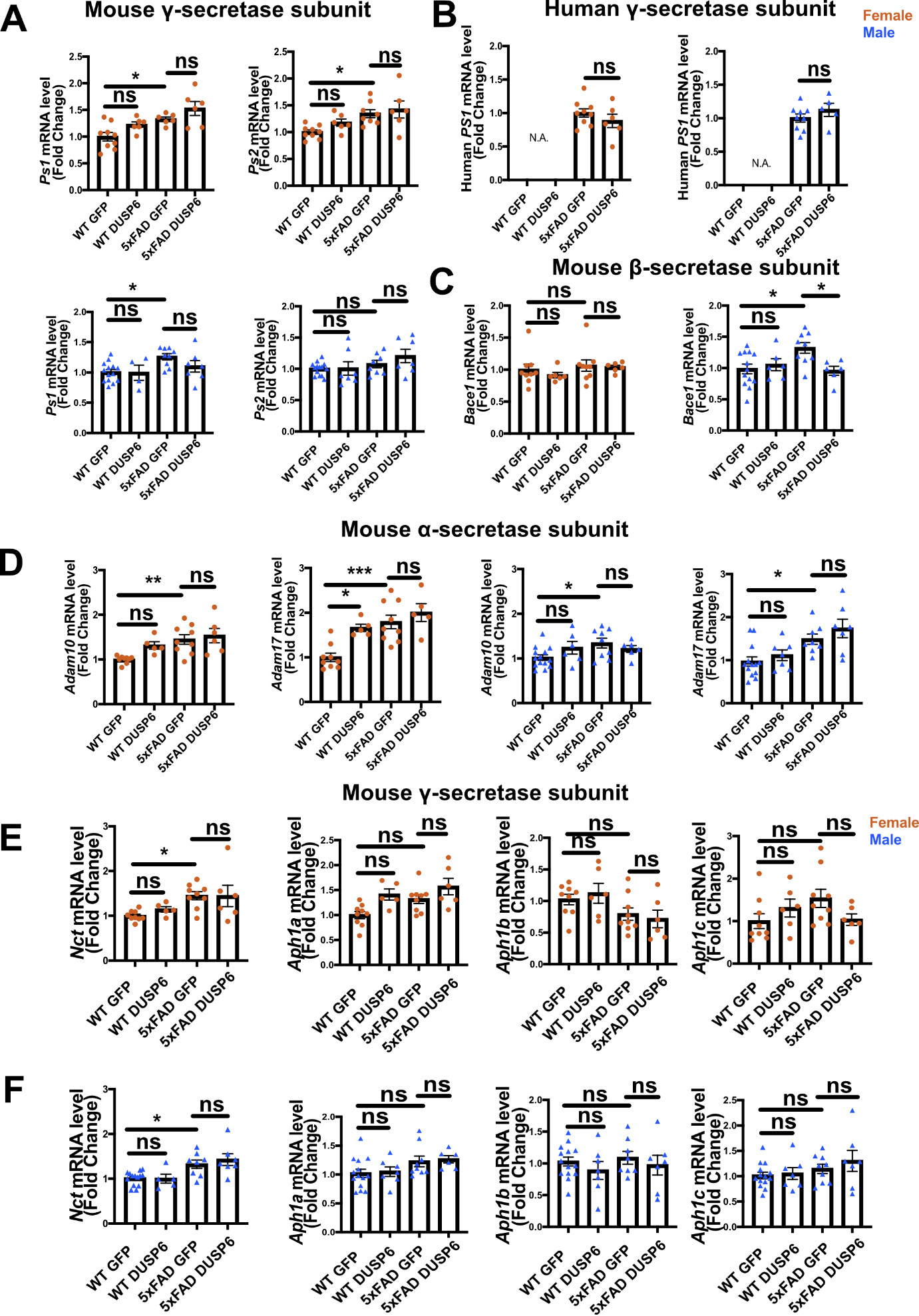


**Fig. S2.** DUSP6 overexpression did not alter mouse APP processing enzyme mRNA levels, other than reduction of BACE1 in male 5xFAD-DUSP6 hippocampus. (A) Hippocampal mouse *Ps1* and *Ps2* mRNAs were assayed from female (top) and male (bottom) 5xFAD and WT mice overexpressing DUSP6 or GFP, n = 6-14 mice per group. (B) Female and male 5xFAD and WT overexpressing DUSP6 or GFP were assayed for human *PS1* mRNA in hippocampus by RT-PCR, n=5-9 mice per group. (C) Female and male 5xFAD and WT overexpressing DUSP6 or GFP were assayed for mouse *Bace1* mRNA in hippocampi by RT-PCR, n = 5-13 mice per group. (D) Female and male 5xFAD and WT overexpressing DUSP6 or GFP were assayed for *Adam10* and *Adam17* mRNAs in hippocampi by RT-PCR. n = 5-14 mice per group. (E, F) Female and male 5xFAD and WT overexpressing DUSP6 or GFP were assayed for mouse *Nct*, *Aph1a*, *Aph1b*, and *Aph1C* mRNAs by RT-PCR, n = 5-14 mice per group. Error bars represent means ± SEM. Statistical analyses were performed using a Student’s t-test for human PS1 mRNA or One-Way ANOVA followed by a Tukey’s post-hoc test for mouse *PS1*, *PS2*, and *Bace1* mRNAs, *p<0.05, **p<0.01, ***p<0.001; N.A. = not applicable, ns = nonsignificant.


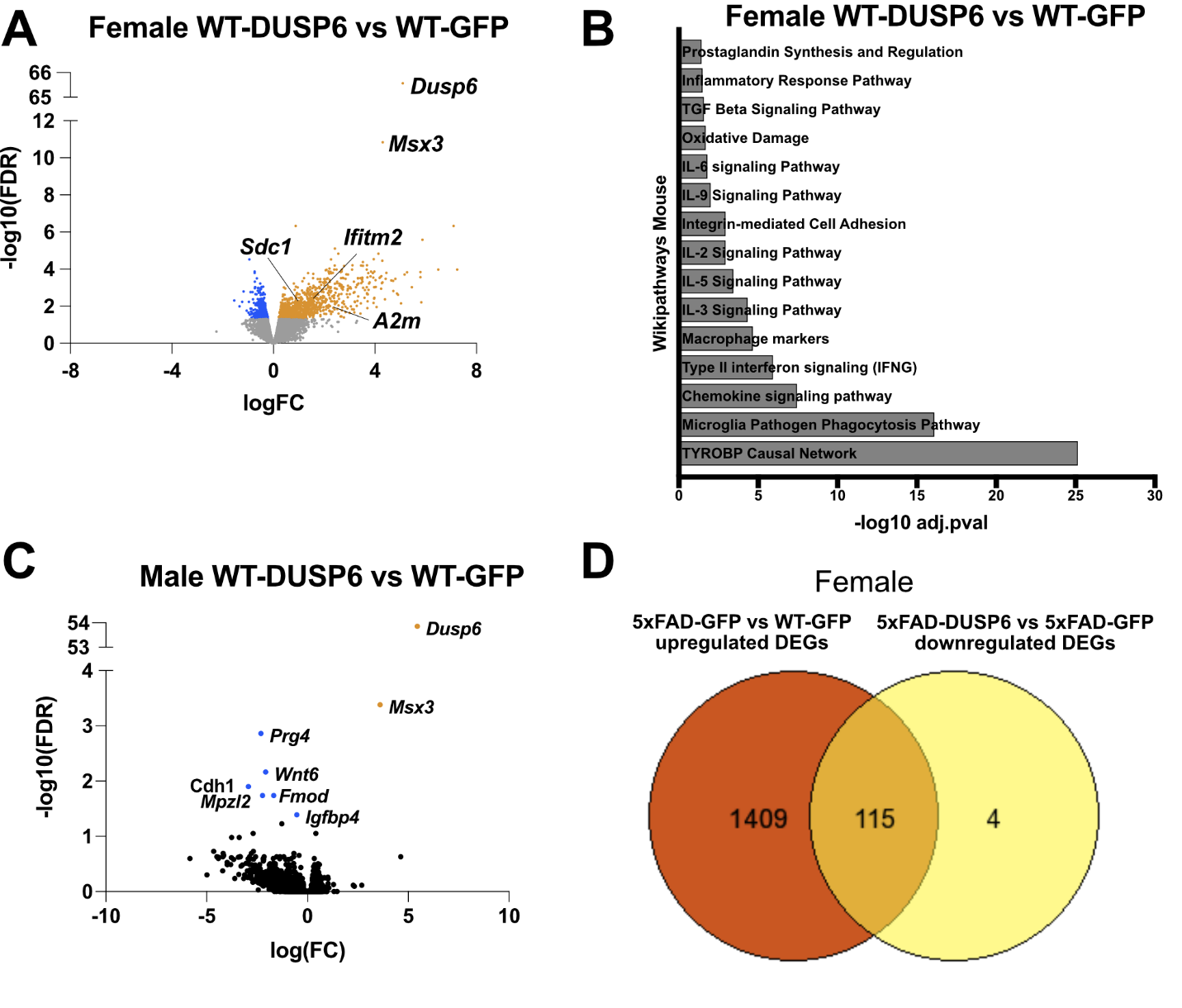


**Fig. S3.** RNA-seq analysis of hippocampi from mice overexpressing DUSP6. (A) Volcano plot representation of DEGs from female WT-DUSP6 vs WT-GFP. (B) Pathway enrichment analysis of the DEGs from A showed inflammatory pathways were involved in WT mice overexpressing DUSP6. (C) Volcano plot representation of DEGs from male WT-DUSP6 vs WT-GFP showed seven DEGs. (D) Venn diagram shows the number of DEGs shared in common among upregulated DEGs from 5xFAD-GFP vs WT-GFP and downregulated genes from 5xFAD-DUSP6 vs 5xFAD-GFP. Orange dots represent upregulated DEGs and blue dots represent downregulated DEGs (FDR < 0.05).

**
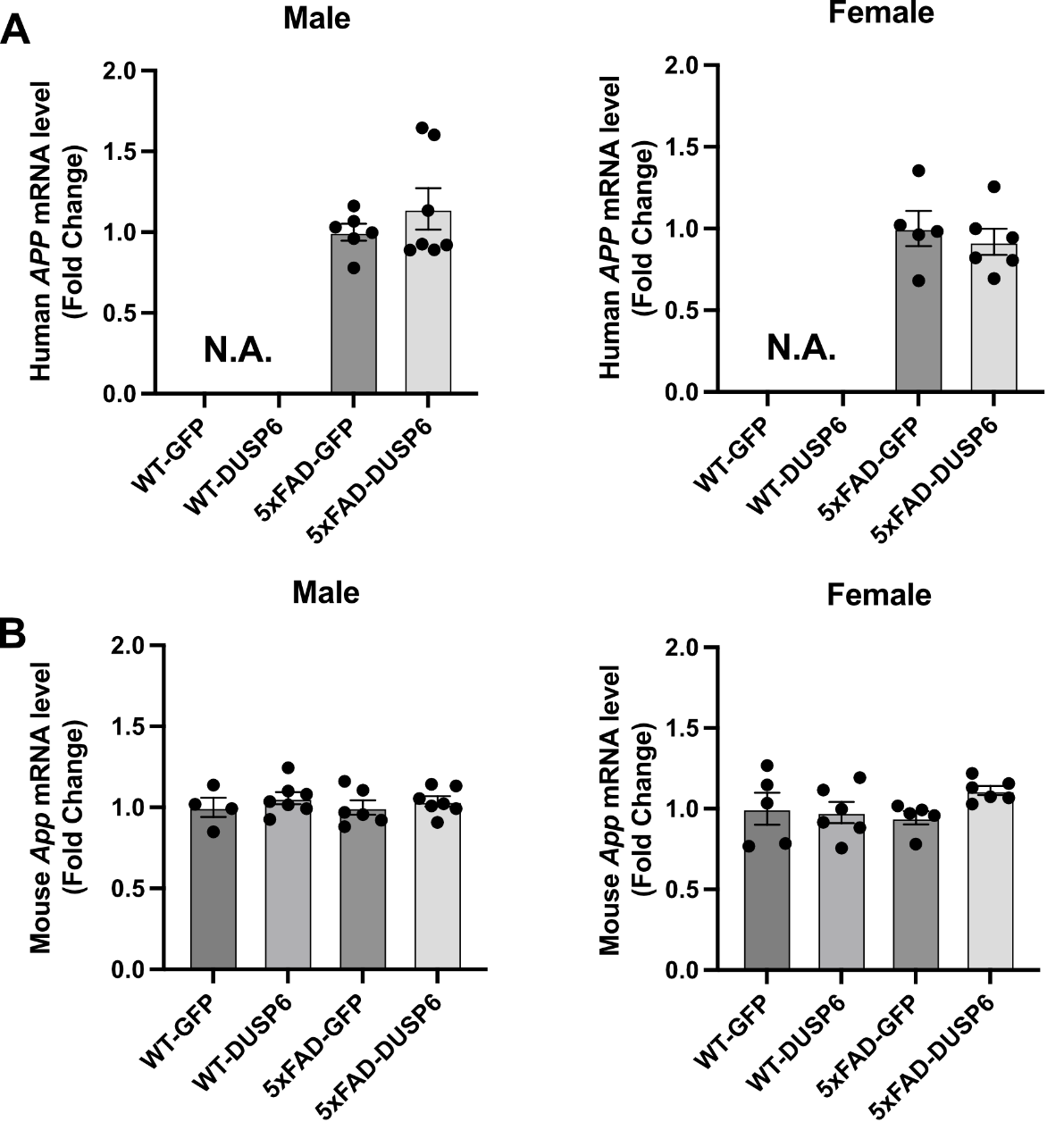
**

**Fig. S4.** RT-qPCR analysis of human *APP* and mouse endogenous *App* mRNA levels in hippocampi from mice overexpressing DUSP6 or GFP. (A) RT-qPCR analyses showed no significant changes of human *APP* mRNA levels in either female or male mice, comparing 5xFAD overexpressing DUSP6 with those overexpressing GFP. N.A. = not applicable. (B) RT-qPCR analyses indicated that overexpression of DUSP6 in either female or male WT (wild type) or 5xFAD did not affect mouse *App* mRNA levels.
